# Marginal bone loss around dental implants: comparison between diabetic and non-diabetic patients—a retrospective clinical study

**DOI:** 10.1007/s00784-023-04872-z

**Published:** 2023-01-30

**Authors:** Sarah Ayele, Nora Sharo, Bruno Ramos Chrcanovic

**Affiliations:** 1 Faculty of Odontology, Malmö University, Malmö, Sweden; 2 Department of Prosthodontics, Faculty of Odontology, Malmö University, Carl Gustafs väg 34, SE-214 21 Malmö, Sweden

**Keywords:** Diabetes, Dental implants, Marginal bone loss, Retrospective clinical study

## Abstract

**Objectives:**

The aim of the present retrospective study was to compare the marginal bone loss (MBL) around dental implants in a group of diabetic patients in relation to a matched group of non-diabetic patients.

**Materials and methods:**

The present dental record–based retrospective study included patients selected from individuals treated with dental implants at one specialist clinic in Malmö, Sweden. Patients were excluded if they had history of periodontitis and/or were treated for periodontal disease. The study group included 710 implants installed in 180 patients (mean age 60.3±13.0 years), 349 implants in 90 diabetic (21 T1DM and 69 T2DM patients), and 361 implants in 90 non-diabetic patients.

**Results:**

The results suggested that jaw (greater MBL in the maxilla), diabetes (greater MBL for diabetic patients, and worse for T1DM patients), bruxism (greater MBL for bruxers), and smoking (greater MBL for smokers and former smokers) had a statistically significant influence on MBL over time.

**Conclusions:**

Patients with diabetes have an estimated greater MBL over time compared to non-diabetic patients. The difference was greater in patients with diabetes type 1 compared to patients with diabetes type 2. Bruxism, smoking, and implant location (maxilla) were also associated with a higher loss of marginal bone around implants over time.

**Clinical relevance:**

Awareness of the possible influence of diabetes on the long-term outcomes of dental implant treatment is important, in order to be able to minimize the possibility of a high MBL with time, which can eventually lead to the loss of the implant.

## Introduction

Diabetes mellitus is a group of metabolic disorders characterized by an inability of the body to produce or properly respond to insulin, resulting in poor preservation of favorable blood glucose levels and thereby hyperglycemia [1]. In type 1 diabetes mellitus (T1DM), the deficiency most commonly occurs due to an auto-immunological destruction of pancreatic insulin-producing β cells. The β cells are insulin-producing; thus, a loss of them results in a complete insulin deficiency [2]. In type 2 diabetes mellitus (T2DM), increased blood glucose levels are a consequence of insulin resistance in cells of muscle and adipose tissue. Due to overnutrition, lack of physical activity, or obesity, compensatory processes for maintenance of the physiological values for blood glucose will be activated in the β cells and will eventually lead to exhaustion of the cells and a dysfunction leading to an inability to secrete sufficient insulin [3, 4].

The long-term hyperglycemia of diabetes mellitus very commonly leads to failure, damage, and/or dysfunction of many tissues and organs of the human body, causing substantial clinical morbidity [5]. A recent article reviewed the possible associations between diabetes and periodontitis, listing evidence suggesting that when uncontrolled, diabetes seems to affect response to periodontal treatment, as well as the risk to develop peri-implant diseases [6]. Although previous research did not show a clear difference in implant failure rates between diabetic and non-diabetic subjects [7–9], another recent review added fresh evidence to the field, suggesting that implants in diabetic patients had a higher failure risk in comparison to non-diabetic patients [10]. This same review identified only a handful of studies looking into the influence of diabetes mellitus on marginal bone loss (MBL), and although there was no clear consensus, the global meta-analytic results suggested worse outcomes for diabetic than for non-diabetic patients.

The subject is important, and dentists need to be made aware of the issue to be able to make a treatment plan aiming to minimize the possibility of a high MBL with time, which can eventually lead to the loss of the implant. The varying results from several studies and the increased use of implants make this an important subject to study for optimal implant installation, healing, and survival. It was therefore the objective of this retrospective study to investigate the influence of diabetes on the MBL surrounding dental implants. The null hypothesis was that there would be no difference in MBL between diabetic and non-diabetic patients, against the alternative hypothesis of a difference.

## Materials and methods

### Objective

The aim of the present retrospective study was to compare the MBL around dental implants in a group of diabetic patients in relation to a matched group of non-diabetic patients. The focused question was elaborated by using the PICO format (Participants, Interventions, Comparisons, Outcomes): “Do diabetic patients undergoing implant-prosthetic rehabilitation present a higher MBL in comparison to non-diabetic patients?”

### Materials

This retrospective study included patients treated with dental implants during the period 1980–2018 at one specialist clinic (Clinic for Prosthodontics, Centre of Dental Specialist Care, Malmö, Sweden). This study was based on data collection from patients’ dental records. The implants were placed by specialist dentists in oral surgery, and dentists performing the prosthetic treatment were specialists in prosthodontics.

The study was approved by the regional Ethical Committee, Lund, Sweden (Dnr 2014/598; Dnr 2015/72). The present retrospective study followed the STROBE guidelines for observational studies and was registered at https://clinicaltrials.gov under the registration number NCT02369562.

### Definitions

Diabetes mellitus was defined according to the International Diabetes Federation^1^ as “a long-term condition that occurs when raised levels of blood glucose occur because the body cannot produce any or enough of the hormone insulin or cannot effectively use the insulin it produces.” Diabetes type 1 applies for cases when the body produces very little or no insulin, and diabetes type 2 for cases when there is an inability of the body’s cells to respond fully to insulin (insulin resistance) [1].

A patient was considered as presenting diabetes when there was information in its records about the intake of oral hypoglycemic drugs or the use of injected insulin, and/or a document from the patient’s medical doctor reporting a diagnosis of the condition.

MBL was defined as loss, in an apical direction, of alveolar bone marginally adjacent to the dental implant, in relation to the marginal bone level initially detected after the implant was surgically placed [11].

As the standard protocol in the clinic, the patients’ dental hygiene was followed up by a dental hygienist within 6 months after the final implant-supported/retained restoration. Each patient then attended a dental hygiene recall program based on individual needs.

### Inclusion and exclusion criteria

All the patients diagnosed as presenting diabetes were included. Only implants not lost and with baseline radiographs taken within 12 months after implant placement and with a minimum of 36 months of radiological follow-up were considered for the analysis of MBL.

Patients with all modern types of threaded implants with cylindrical or conical design were included. Zygomatic implants were not included in the study, as well as implants detected in radiographies, but without basic information about them in the patients’ files.

Patients were excluded if they had a history of periodontitis and/or were treated for periodontal disease. It is important to take note that as standard, all patients receiving implants at the Specialist Clinic for Prosthodontics were periodontally healthy at the time of implant installation. Patients with either a history or with signs of periodontal disease were treated at the Specialist Clinic for Periodontology, where they later could or not receive dental implants, according to individual needs/indications. These patients were not included in the present study.

### Data collection

The data were directly entered into an SPSS file (SPSS software, version 28, SPSS Inc., Chicago, IL, USA) as the dental records of the patients were being read, and it consisted of several implant-, site-, and patient-related factors.

### Formation of a matched group

Since the division of all initial patients into groups would generate extremely unbalanced groups and the variance was not homogenous between them, the two groups were therefore not expected to be comparable with respect to important covariates [12], and then methods were used to match patients and implants between diabetic to non-diabetic patients. Matching ensures that any differences between the study and the control groups are not a result of differences in the matching variables, thus reducing selection bias.

The matching was performed using the “case control matching” function in SPSS, and the matches were selected on the basis of similarities in (a) patients’ age at the time of the surgery, (b) number of implants, and (c) total radiological follow-up time. As there were no perfect matches in a first matching attempt considering all three variables, some tolerance was set for the predictors: ± 5 years for the patients’ age, ± 2 implants, and ± 12 months for the total radiological follow-up time. Thus, a little variance of these predictors between the groups was expected.

### Marginal bone level evaluation

The evaluation of the variation of the marginal bone level over time was performed according to a previous study [13]. Reproducible intra-oral radiographs were used. When there were no available digital radiographies from the baseline appointment, the analogue periapical radiographies were scanned at 1200 dpi (Epson Perfection V800 Photo Color Scanner; Nagano, Japan). Marginal bone level (MBL) was measured after calibration based on the inter-thread distance of the implants. Measurements were taken from the implant-abutment junction to the marginal bone level, at both mesial and distal sides of each implant, and then the mean value of these two measurements was considered. MBL was calculated by comparing bone-to-implant contact levels to the radiographic baseline examination. The ImageJ software (National Institute of Health, Bethesda, USA) was used for all measurements. Negative values of MBL corresponded to bone loss.

The sets of radiographs for every patient were codified and the authors who performed the radiological measurements (S. A., N. S.) were blinded to the diagnosis of the condition for every patient.

### Calibration

An initial calibration concerning MBL was performed between the authors. The process was done for 10 random samples from the cohort group and verified after the measurement of each sample. At the end of the process, the measurements from the different individuals were considered enough approximate from each other, with agreement between examiners set at > 90% of the distance in millimeters.

### Sample size calculation

A calculation of the sample size was not conducted. The reason is that the database from which the eligible cases for the present study were originated had a certain number of patients and dental implants, namely approximately 2800 and 11,000 respectively, and it would not possible to recruit more cases, as the database already included all patients treated with dental implants during the aforementioned period in the specialist clinic.

Instead, all the diabetic patients were initially considered eligible for inclusion, in order to get the maximum number of cases available, namely the largest sample size possible from this database, provided that these cases would fulfill the inclusion criteria, i.e., baseline radiographs taken within 12 months after implant placement and with a minimum of 36 months of radiological follow-up. The number of implants in the diabetic patients was then matched to a group of non-diabetic patients.

### Statistical analyses

The mean, standard deviation, and percentages were presented as descriptive statistics. Kolmogorov–Smirnov test was performed to evaluate the normal distribution of the variables, and Levene’s test evaluated homoscedasticity. The performed tests for two independent groups were Student’s *t*-test or Mann-Whitney test, and for three or more independent groups were ANOVA or Kruskal-Wallis test, depending on the normality. Paired *t*-test or Wilcoxon’s signed-rank test was used to compare the mean value difference of continuous variables between dependent groups. Pearson’s chi-squared test or Fisher’s exact test was used in the analysis of contingency tables of categorical data of independent groups, and McNemar’s test for dependent groups.

Univariate linear regression models were used to compare MBL over time between clinical covariates. In order to verify multicollinearity, a correlation matrix of all of the predictor variables was scanned, to see whether there were some high correlations among the predictors. Collinearity statistics obtaining variance inflation factor (VIF) and tolerance statistics were also performed to detect more subtle forms of multicollinearity. A linear mixed-effects model was built with all variables that were moderately associated (*p* < 0.10) with MBL in the univariate linear regression models. A mixed-effects model was used in order to take into consideration that some patients had more than one implant-supported prosthesis, as multiple observations within an individual are not independent of each other.

The degree of statistical significance was considered *p* < 0.05. Data were statistically analyzed using the Statistical Package for the Social Sciences (SPSS) version 28 software (SPSS Inc., Chicago, IL, USA).

## Results

The cohort group included 710 implants installed in 180 patients. There were 349 implants installed in 90 diabetic patients, of which 54 implants were in 21 T1DM patients and 295 implants in 69 T2DM patients. There were 361 implants installed in 90 non-diabetic patients.

The mean age (± SD) of the 180 patients was 60.3 ± 13.0 years (min–max, 15.2–85.5) at the day of the implant surgical placement. The patients were followed up clinically for a mean (± SD) of 140.2 ± 76.4 months (min–max, 36.7–381.8), and radiographically for a mean (± SD) of 110.2 ± 69.0 months (min–max, 36.7–363.0).

Table 1 shows the descriptive data of the implants included in the study. The variable patient’s age was divided into three categories each, based on the 33.3 and 66.7 percentiles of sample distribution, in order to generate groups of more balanced sample sizes. There was a difference in the distribution of implants of different surfaces between the groups, as well as for the type of prosthesis the implants were supporting.

**Table 1:** Descriptive data of the implants included in the study, separated by group. The statistical unit is the implant, not the patient

Factor	Diabetic, implants (%)	Non-diabetic, implants (%)	*p* value
*Follow-up*^*a*^ *(months)*			
(mean ± SD)	115.1 ± 75.4	105.7 ± 60.1	0.075^b^
*Age*			
(mean ± SD)	59.3 ± 12.7	61.3 ± 13.2	0.060^b^
*Sex*			
Male	198 (56.7)	219 (60.7)	0.287^c^
Female	151 (43.3)	142 (39.3)	
*Age (years)*			
< 58	124 (35.5)	113 (31.3)	0.434^c^
58–67	111 (31.8)	128 (35.5)	
> 67	114 (32.7)	120 (33.2)	
*Jaw*			
Maxilla	183 (52.4)	209 (57.9)	0.101^c^
Mandible	166 (47.6)	152 (42.1)	
*Jaw region*			
Anterior	189 (54.2)	174 (48.2)	0.113^c^
Posterior	160 (45.8)	187 (51.8)	
*Tooth region*			
Incisor	119 (34.1)	105 (29.1)	0.425^c^
Canine	70 (20.0)	69 (19.1)	
Premolar	129 (37.0)	150 (41.6)	
Molar	31 (8.9)	37 (10.2)	
*Implant diameter*			
3.00–3.50 mm	32 (9.2)	22 (6.1)	0.283^c^
3.75–4.10 mm	310 (88.8)	330 (91.4)	
4.30–5.00 mm	7 (2.0)	9 (2.5)	
*Implant surface*			
Turned	171 (49.0)	79 (21.9)	< 0.001^c^
Modified	178 (51.0)	282 (78.1)	
*Prosthesis type*^b^			
Single crown	27 (7.8)	43 (11.9)	< 0.001^c^
FDP 2–6 units	119 (34.5)	156 (43.2)	
FDP 7–10 units	15 (4.3)	36 (10.0)	
Full-arch	181 (52.5)	126 (34.9)	
Overdenture	3 (0.9)	0 (0.0)	
*Prosthesis fixation*^b^			
Cemented	24 (6.9)	21 (5.9)	0.595^c^
Screwed	325 (93.1)	335 (94.1)	
*Bruxism*^d^			
No	272 (80.7)	290 (80.3)	0.899^c^
Yes	65 (19.3)	71 (19.7)	
*Smoking*^d^			
No	221 (67.8)	249 (69.0)	0.328^c^
Yes	92 (28.2)	90 (24.9)	
Former smokers	13 (4.0)	22 (6.1)	

^a^Radiological follow-up

^b^Wilcoxon’s signed-rank test

^c^Comparison of the distribution of implants, among the categories of each factor, between diabetic and non-diabetic patients

^d^For the cases with available information

The total number of marginal bone level double measurements (mesial + distal sides of each implant) was 4555, with 1972 double measurements in diabetic and 2583 in non-diabetic patients. Table 2 shows data on MBL distributed by different periods of follow-up, separated by groups of patients. Implants in T1DM patients generally showed higher MBL for the same periods of follow-up in comparison to T2DM patients, the same observed with T2DM patients in comparison to non-diabetic patients. It is important to stress that not all implants in all patients were followed up for the same period of time.

**Table 2:** Data on marginal bone loss distributed by different periods of follow-up, separated per groups of patients. Values in millimeters. Negative values correspond to bone loss

Follow-up	T1DM patients	T2DM patients	Non-diabetic patients
		Mean ± SD (min, max)	
0–1 year	−0.16 ± 0.26 (−1.34, 0.158)	−0.14 ± 0.27 (−1.74, 0.82)	−0.11 ± 0.25 (−0.99, 0.90)
1–2 years	−1.08 ± 0.40 (−2.51, −0.35)	−0.50 ± 0.38 (−1.51, 0.53)	−0.36 ± 0.38 (−1.98, 0.55)
2–3 years	−1.05 ± 0.65 (−3.68, −0.32)	−0.74 ± 0.41 (−1.78, −0.04)	−0.53 ± 0.44 (−1.99, 0.66)
3–4 years	−1.77 ± 0.67 (−4.33, −0.96)	−0.96 ± 0.53 (−2.42, 0.08)	−0.51 ± 0.42 (−1.87, 0.70)
4–5 years	−1.68 ± 0.68 (−3.02, −0.98)	−1.26 ± 0.64 (−2.90, 0.54)	−0.68 ± 0.60 (−3.03, 0.94)
5–10 years	−2.28 ± 0.76 (−4.87, −0.18)	−1.47 ± 0.82 (−4.39, 0.49)	−0.91 ± 0.65 (−3.01, 0.81)
10–15 years	−3.34 ± 0.94 (−5.49, −1.84)	−1.83 ± 0.84 (−5.93, −0.48)	−1.18 ± 0.72 (−2.72, 0.22)
15–30 years	−2.60 ± 0.50 (−3.15, −1.58)	−2.29 ± 0.89 (−6.06, −1.02)	−1.74 ± 1.02 (−4.07, 0.19)

*T1DM* type 1 diabetes mellitus

*T2DM* type 2 diabetes mellitus

*SD* standard deviation

**Fig. 1:**
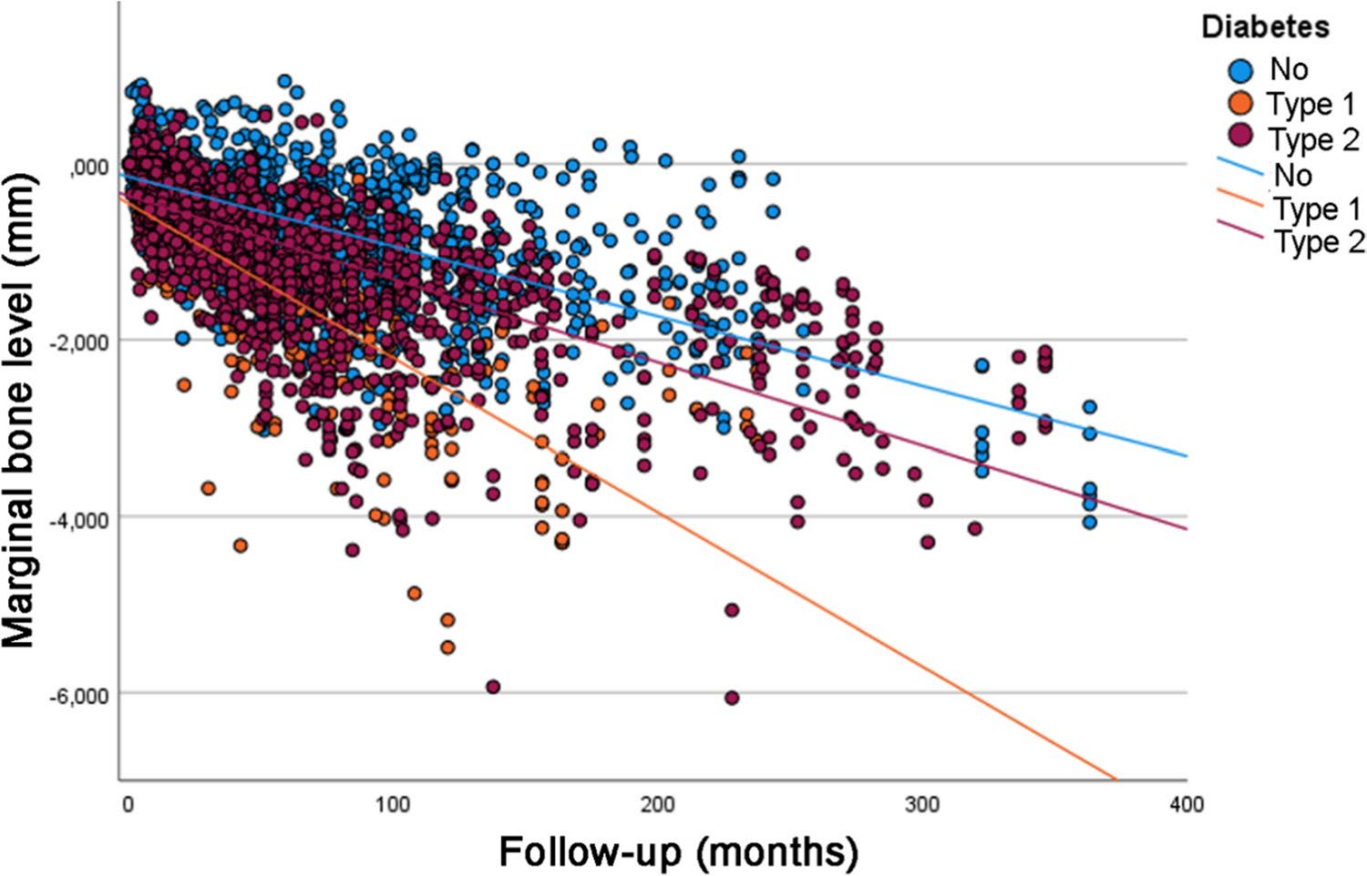
Scatter plot comparing the marginal bone level over time between implants placed in diabetic (T1DM and T2DM) and non-diabetic patients (linear regression)

**Fig. 2:**
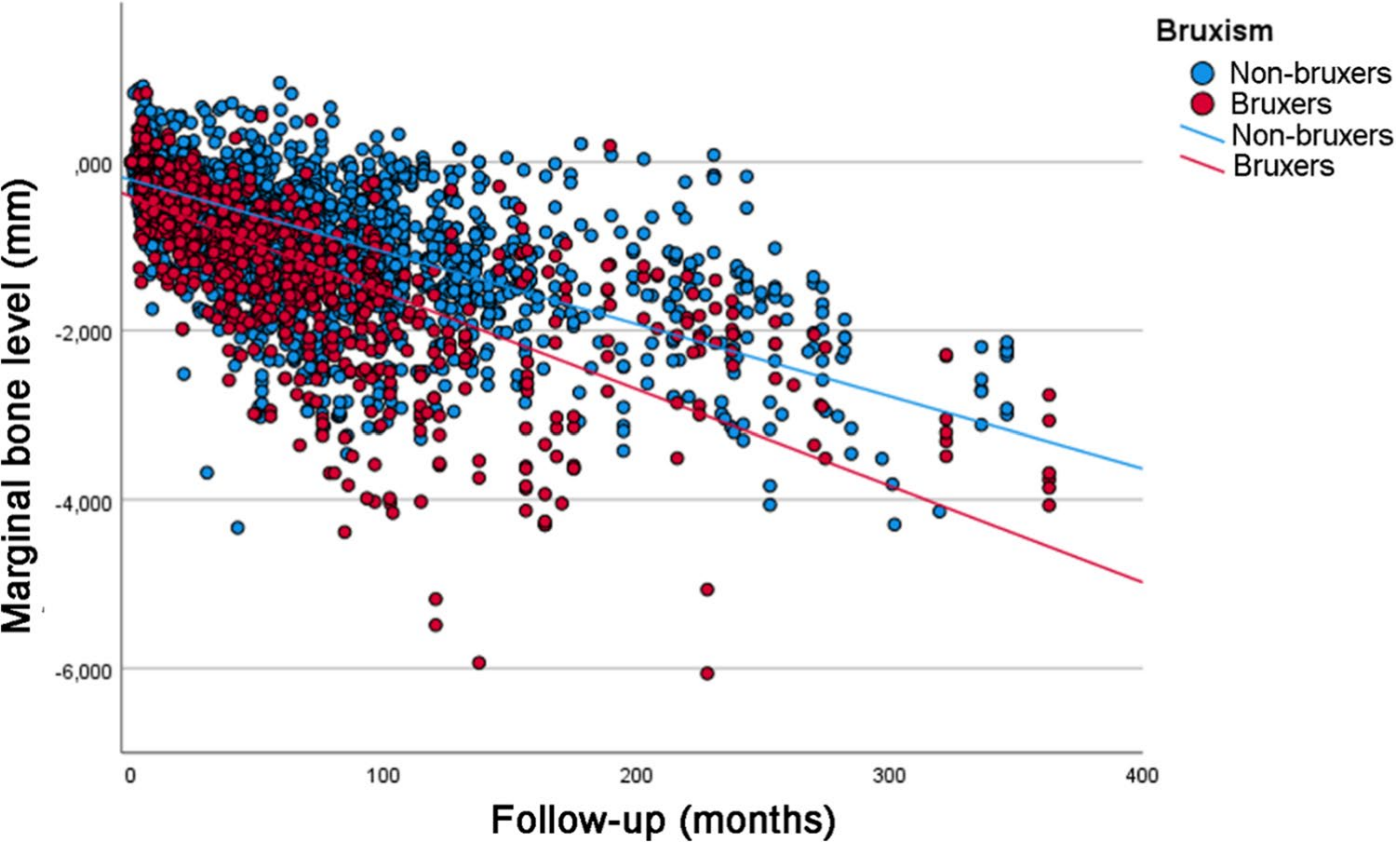
Scatter plot comparing the marginal bone level over time between implants placed in bruxers and non-bruxer patients (linear regression)

**Fig. 3:**
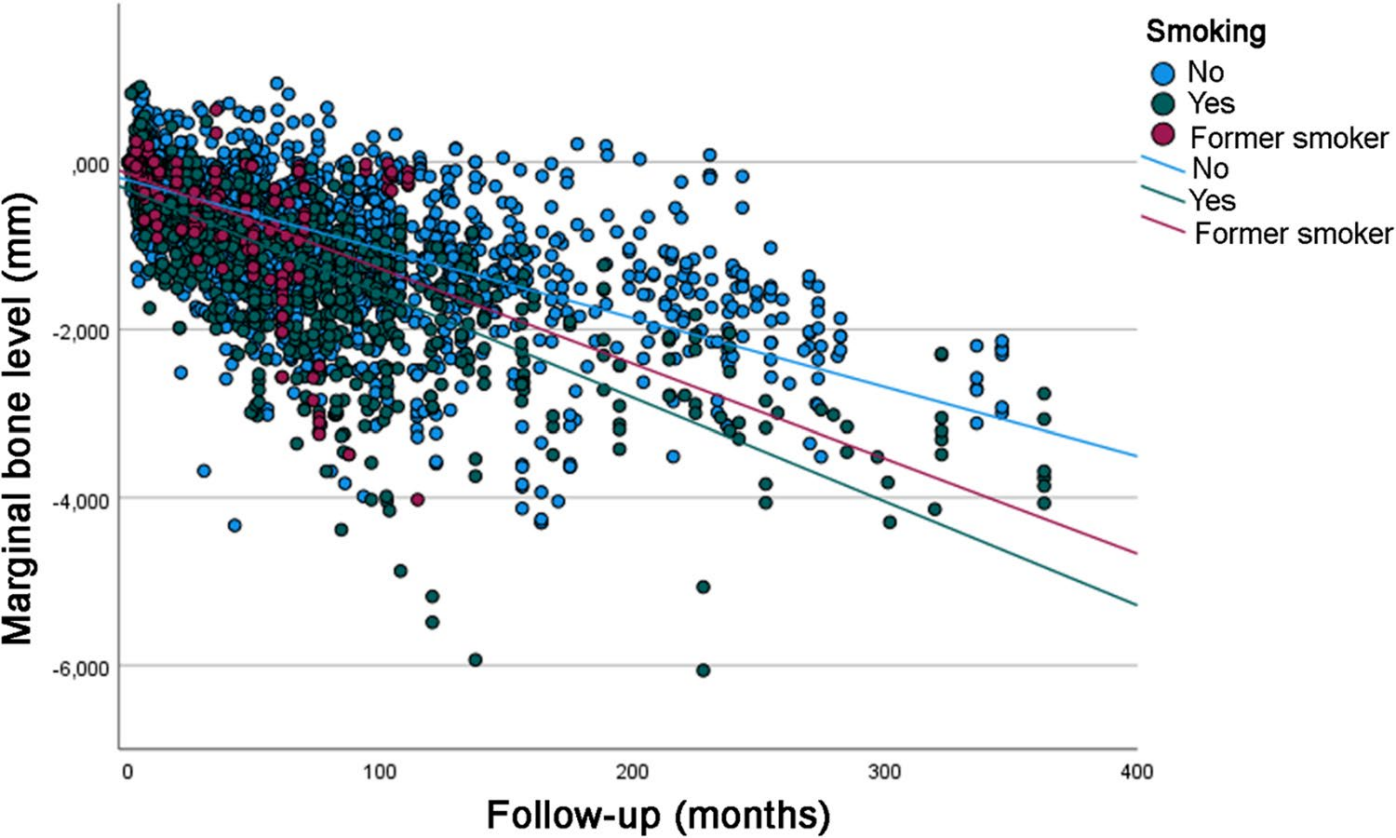
Scatter plot comparing the marginal bone level over time between implants placed in smokers, former smokers, and non-smokers (linear regression)

Analysis of the univariate linear regression analysis (Table 3) showed that the estimated MBL over time was statistically significantly different between the categories of the following variables: patient’s age at the time of implant placement, jaw (greater MBL in the maxilla), implant diameter (greater MBL for wide diameter implants), implant surface (greater MBL for implants with modified surface), prosthesis type (greater MBL for overdentures), diabetes (greater MBL for diabetic patients, and worse for T1DM patients) (Fig. 1), bruxism (greater MBL for bruxers) (Fig. 2), and smoking (greater MBL for smokers and former smokers) (Fig. 3). Most of the categories had a moderate degree of linear correlation (*R*^2^ linear) with MBL over time.

**Table 3:** Univariate linear regression analysis for MBL

Factor	Linear equation*	*p* value ^a^	*R*^2^ linear
*Sex*			
Male	*y* = *−*0.23 − 0.00947*x*	0.435	0.449
Female	*y* = *−*0.26 − 0.00974*x*		0.470
*Age (years)*			
< 58	*y* = *−*0.29 − 0.00991*x*	< 0.001	0.518
58–67	*y* = *−*0.29 − 0.00874*x*		0.380
> 67	*y* = *−*0.16 − 0.00937*x*		0.394
*Jaw*			
Maxilla	*y* = *−*0.21 − 0.01000*x*	< 0.001	0.480
Mandible	*y* = *−*0.26 − 0.00866*x*		0.449
*Jaw region*			
Anterior	*y* = *−*0.21 − 0.01000*x*	0.324	0.481
Posterior	*y* = *−*0.27 − 0.00864*x*		0.445
*Tooth region*			
Incisor	*y* = *−*0.22 − 0.01000*x*	0.332	0.492
Canine	*y* = *−*0.18 − 0.00991*x*		0.489
Premolar	*y* = *−*0.27 − 0.00905*x*		0.447
Molar	*y* = *−*0.27 − 0.00742*x*		0.455
*Implant diameter*			
3.00–3.50 mm	*y* = *−*0.17 − 0.00917*x*	0.049	0.387
3.75–4.10 mm	*y* = *−*0.25 − 0.00951*x*		0.459
4.30–5.00 mm	*y* = *−*0.04 − 0.02000*x*		0.554
*Implant surface*			
Turned	*y* = *−*0.29 − 0.00858*x*	< 0.001	0.464
Modified	*y* = *−*0.17 − 0.01000*x*		0.435
*Prosthesis type*			
Single crown	*y* = *−*0.16 − 0.01000*x*	< 0.001	0.477
FDP 2–6 units	*y* = *−*0.26 − 0.00850*x*		0.455
FDP 7–10 units	*y* = *−*0.21 − 0.01000*x*		0.534
Full-arch	*y* = *−*0.21 − 0.01000*x*		0.476
Overdenture	*y* = *−*0.58 − 0.03000*x*		0.206
*Prosthesis fixation*^*b*^			
Cemented	*y* = *−*0.14 − 0.01000*x*	0.069	0.620
Screwed	*y* = *−*0.25 − 0.00956*x*		0.460
*Diabetes*			
No	*y* = *−*0.15 − 0.00791*x*	< 0.001	0.442
Type 2	*y* = *−*0.37 − 0.00944*x*		0.625
Type 1	*y* = *−*0.46 − 0.02000*x*		0.492
*Bruxism*^b^			
No	*y* = *−*0.22 − 0.00854*x*	< 0.001	0.444
Yes	*y* = *−*0.41 − 0.01000*x*		0.507
*Smoking*^b^			
No	*y* = *−*0.22 − 0.00821*x*	< 0.001	0.428
Former smoker	*y* = *−*0.15 − 0.01000*x*		0.589
Yes	*y* = *−*0.34 − 0.01000*x*		0.234

*For the linear equation, “*x*” represents the number of months

^a^Comparison of the slope of the equation (variation of MBL in mm in time) between groups

^b^For the cases with available information

The results of the linear mixed-effects model (Table 4) suggested that jaw (greater MBL in the maxilla), diabetes (greater MBL for diabetic patients, and worse for T1DM patients), bruxism (greater MBL for bruxers), and smoking (greater MBL for smokers and former smokers) had a statistically significant influence on MBL over time.

**Table 4:** Linear mixed-effects model for MBL

Predictor variables	*F* statistic	*p* value
Age	2.551	0.075
Jaw	6.561	< 0.001
Implant diameter	0.308	0.899
Implant surface	3.768	0.055
Prosthesis type	1.356	0.295
Diabetes	37.277	< 0.001
Bruxism	102.855	< 0.001
Smoking	22.987	< 0.001

## Discussion

The aim of the present retrospective study was to investigate the influence of diabetes mellitus on MBL around dental implants. According to the results of the present study, there was a statistically significant difference in MBL over time between non-diabetic and diabetic patients, in particular in patients with T1DM. Therefore, the null hypothesis was rejected.

These results can be explained by the negative effects of diabetic-associated hyperglycemia, which causes excessive secretion of pro-inflammatory cytokines and thereby increased osteoclastic bone resorption [14, 15]. Impaired healing is a consequence of diabetic conditions which could possibly jeopardize the initial bone-to-implant contact (BIC). This could also be linked to a higher MBL since an impaired healing response and less BIC could affect the ability of the implant to resist loading and bacterial infection [16, 17].

There is a difference in the cause of the hyperglycemic condition in subjects with type 1 versus type 2 diabetes. In subjects with T1DM, there is a complete insulin deficiency whereas in T2DM subjects’ insulin secretion gradually decreases, due to fatigued β cells. Therapy of the condition varies between the two. In T1DM, patients are insulin-dependent, meaning they have continuous administration of insulin during the day. T2DM patients can usually be treated in several ways, for instance with oral drugs such as metformin and/or lifestyle and diet changes [18]. A study that examined the two groups and their ability to maintain the set therapeutic blood glucose value showed that fewer children and adolescents with T1DM were able to maintain target levels. This would then mean that T1DM are more likely to be in a hyperglycemic state and therefore be exposed to the bone-related consequences that follow [19].

This may be connected to the fact that diabetes can have a negative impact on bone metabolism and healing. This is due to an increased intensity and constancy of inflammatory infiltrate, elevated osteoclast activity, and increased apoptosis of osteoblasts. When stimulated, osteoblasts express receptor activators of nuclear factor-kappa B ligand (RANKL), which binds to osteoclasts via its receptor (RANK) to activate osteoclastic production of acids and acid hydrolases [20]. The acidic production results in the destruction of the bone. Activation of osteoclasts can be inhibited by the binding of RANKL to a decoy receptor osteoprotegerin (OPG) [21]. Diabetic hyperglycemia may alter the RANKL:OPG ratio, leading to a greater osteoclastic activity and thereby increased bone loss [22]. This could possibly lead to some influence on MBL around dental implants in diabetic patients. An animal study observed that, in relation to a normoglycemic group, diabetes caused a more persistent inflammatory response in ligature-induced periodontitis model, greater loss of attachment and more alveolar bone resorption, and impaired new bone formation [23]. In another study, diabetic animals produced sufficient amounts of immature mesenchymal tissue but failed to adequately express genes that regulate osteoblast differentiation, which in turn, leads to decreased bone formation [24].

Our result presented a statistically significant difference in MBL in patients with bruxism in comparison to patients without bruxism. According to an international consensus, bruxism is defined as “repetitive masticatory muscle activity characterized by clenching or grinding of the teeth and/or by bracing or thrusting of the mandible and specified as either sleep bruxism or awake bruxism” [25]. The occlusal force produced in patients with bruxism could possibly exert an excessive load on the implant-supported crown and its fixture. One can assume that this also would have an effect on the peri-implant bone and thereby the marginal bone. The findings of some studies have shown that bruxism may be a significant factor resulting in an increase in implant failure rate [26–28], an increased incidence of a series of technical complications [28–31], and even implant fracture [32]. Regarding MBL, it was concluded that occlusal overloading had a negative effect on MBL [33]. The results of the first clinical study comparing MBL in a group of bruxers in relation to a matched group of non-bruxers suggested that bruxism increases the risk of MBL over time [13].

The present results indicated a difference in MBL around implants between smoking and non-smoking subjects. These findings are consistent with previous studies. Systematic reviews and meta-analyses concluded that smoking had a negative effect on implant failure rates and MBL around implants compared to non-smokers [34, 35]. This can be explained by the negative effects smoking has on bone metabolism. Smoking reduces the rate of bone mineralization and thickness in bone trabeculae, which consequently causes a decrease in bone formation [36]. It has also been shown that cigarette smoke influences the activation and differentiation of osteoclasts, leading to an increase in bone resorption [37]. This is due to elevated levels of oxidative stress and free radicals seen in smokers [38, 39]. Moreover, smoking affects the process of angiogenesis negatively [40, 41]. Angiogenesis is a process through which new blood cells are formed from pre-existing vessels, which is necessary for healing and in the process of osseointegration of implants [42]. Thus, impaired angiogenesis due to smoking can affect bone repair and bone metabolism negatively.

When it comes to the site of the implant, our results show a difference between the maxilla and mandible, with a greater MBL in the maxilla. A possible explanation for this could be the difference in bone density between the jaws. One study showed that the cortical bone of the mandible showed a higher density when compared to the cortical bone of the maxilla [43]. When examining differences in bone density, one study concluded that the lower jaw also showed a higher density in trabecular bone [44]. In another study, the association between MBL and bone quality around implants was examined. It was concluded that increased bone quality, meaning bone with higher density of trabecular and thick or thin cortical bone, was associated with a decrease in bone loss [45]. Bone sites with lower bone quality have also been implicated in higher dental implant failures [46].

As limitations of the present study, this was a dental record–based retrospective study. The nature of a retrospective study inherently results in flaws. These problems were manifested by the gaps in information and incomplete records. Furthermore, all data rely on the accuracy of the original examination and documentation. Items may have been excluded in the initial examination or not recorded in the dental chart.

As conclusions, the results of the present study suggest the following: (a) patients with diabetes present greater MBL around implants over time compared to non-diabetic patients, (b) the difference was greater in patients with diabetes type 1 (T1DM) compared to patients with diabetes type 2 (T2DM), and (c) other factors that have a negative impact on MBL are smoking and bruxism.

## References

[1] IDF (2021). International Diabetes Federation.

[2] Bluestone JA, Herold K, Eisenbarth G (2010). Genetics, pathogenesis and clinical interventions in type 1 diabetes. Nature.

[3] Cavaghan MK, Ehrmann DA, Polonsky KS (2000). Interactions between insulin resistance and insulin secretion in the development of glucose intolerance. J Clin Invest.

[4] Kahn BB, Flier JS (2000). Obesity and insulin resistance. J Clin Invest.

[5] Reenders K, de Nobel E, van den Hoogen HJ, Rutten GE, van Weel C (1993). Diabetes and its long-term complications in general practice: a survey in a well-defined population. Fam Pract.

[6] Nibali L, Gkranias N, Mainas G, Di Pino A (2022). Periodontitis and implant complications in diabetes. Periodontol 2000.

[7] Chrcanovic BR, Albrektsson T, Wennerberg A (2014). Diabetes and oral implant failure: a systematic review. J Dent Res.

[8] Chrcanovic BR, Kisch J, Albrektsson T, Wennerberg A (2016). Factors influencing early dental implant failures. J Dent Res.

[9] Chrcanovic BR, Kisch J, Albrektsson T, Wennerberg A (2018). A retrospective study on clinical and radiological outcomes of oral implants in patients followed up for a minimum of 20 years. Clin Implant Dent Relat Res.

[10] Al Ansari Y, Shahwan H, Chrcanovic BR (2022). Diabetes mellitus and dental implants: a systematic review and meta-analysis. Materials (Basel).

[11] Albrektsson T, Chrcanovic B, Östman PO, Sennerby L (2017). Initial and long-term crestal bone responses to modern dental implants. Periodontol 2000.

[12] D'Agostino RB (1998). Propensity score methods for bias reduction in the comparison of a treatment to a non-randomized control group. Stat Med.

[13] Bredberg C, Vu C, Haggman-Henrikson B, Chrcanovic BR (2022) Marginal bone loss around dental implants: comparison between matched groups of bruxer and non-bruxer patients: a retrospective case-control study. Clin Implant Dent Relat Res. 10.1111/cid.13161 10.1111/cid.13161 PMC10099792 36411179

[14] Jiang X, Zhu Y, Liu Z, Tian Z, Zhu S (2021). Association between diabetes and dental implant complications: a systematic review and meta-analysis. Acta Odontol Scand.

[15] Souto-Maior JR, Pellizzer EP, de Luna Gomes JM, Dds C, Dds J, Vasconcelos B, de Moraes SLD (2019). Influence of diabetes on the survival rate and marginal bone loss of dental implants: an overview of systematic reviews. J Oral Implantol.

[16] Accursi GE (2000). Treatment outcomes with osseointegrated Brånemark implants in diabetic patients: a retrospective study.

[17] Takeshita F, Murai K, Iyama S, Ayukawa Y, Suetsugu T (1998). Uncontrolled diabetes hinders bone formation around titanium implants in rat tibiae. a light and fluorescence microscopy, and image processing study. J Periodontol.

[18] Watkins PJ (2003). ABC of diabetes.

[19] Van Name MA, Cheng P, Gal RL, Kollman C, Lynch J, Nelson B, Tamborlane WV, Pediatric Diabetes C (2020). Children and adolescents with type 1 and type 2 diabetes mellitus in the Pediatric Diabetes Consortium Registries: comparing clinical characteristics and glycaemic control. Diabet Med.

[20] Tatsumi S, Ishii K, Amizuka N, Li M, Kobayashi T, Kohno K, Ito M, Takeshita S, Ikeda K (2007). Targeted ablation of osteocytes induces osteoporosis with defective mechanotransduction. Cell Metab.

[21] Boyce BF, Xing L (2007). The RANKL/RANK/OPG pathway. Curr Osteoporos Rep.

[22] Yoshida T, Flegler A, Kozlov A, Stern PH (2009). Direct inhibitory and indirect stimulatory effects of RAGE ligand S100 on sRANKL-induced osteoclastogenesis. J Cell Biochem.

[23] Liu R, Bal HS, Desta T, Krothapalli N, Alyassi M, Luan Q, Graves DT (2006). Diabetes enhances periodontal bone loss through enhanced resorption and diminished bone formation. J Dent Res.

[24] Lu H, Kraut D, Gerstenfeld LC, Graves DT (2003). Diabetes interferes with the bone formation by affecting the expression of transcription factors that regulate osteoblast differentiation. Endocrinology.

[25] Lobbezoo F, Ahlberg J, Raphael KG, Wetselaar P, Glaros AG, Kato T, Santiago V, Winocur E, De Laat A, De Leeuw R, Koyano K, Lavigne GJ, Svensson P, Manfredini D (2018). International consensus on the assessment of bruxism: report of a work in progress. J Oral Rehabil.

[26] Chrcanovic BR, Albrektsson T, Wennerberg A (2015). Bruxism and dental implants: a meta-analysis. Implant Dent.

[27] Chrcanovic BR, Kisch J, Albrektsson T, Wennerberg A (2016). Bruxism and dental implant failures: a multilevel mixed effects parametric survival analysis approach. J Oral Rehabil.

[28] Chrcanovic BR, Kisch J, Albrektsson T, Wennerberg A (2017). Bruxism and dental implant treatment complications: a retrospective comparative study of 98 bruxer patients and a matched group. Clin Oral Implants Res.

[29] Chrcanovic BR, Kisch J, Larsson C (2020). Retrospective clinical evaluation of 2- to 6-unit implant-supported fixed partial dentures: mean follow-up of 9 years. Clin Implant Dent Relat Res.

[30] Chrcanovic BR, Kisch J, Larsson C (2020). Retrospective evaluation of implant-supported full-arch fixed dental prostheses after a mean follow-up of 10 years. Clin Oral Implants Res.

[31] Chrcanovic BR, Kisch J, Larsson C (2020). Analysis of technical complications and risk factors for failure of combined tooth-implant-supported fixed dental prostheses. Clin Implant Dent Relat Res.

[32] Chrcanovic BR, Kisch J, Albrektsson T, Wennerberg A (2018). Factors influencing the fracture of dental implants. Clin Implant Dent Relat Res.

[33] Fu JH, Hsu YT, Wang HL (2012). Identifying occlusal overload and how to deal with it to avoid marginal bone loss around implants. Eur J Oral Implantol.

[34] Chrcanovic BR, Albrektsson T, Wennerberg A (2015). Smoking and dental implants: a systematic review and meta-analysis. J Dent.

[35] Mustapha AD, Salame Z, Chrcanovic BR (2021). Smoking and dental implants: a systematic review and meta-analysis. Medicina (Kaunas).

[36] Barbosa AP, Lourenço JD, Junqueira JJM, Emidio L, de França S, Martins JS, Oliveira Junior MC, Begalli I, Velosa APP, Olivo CR, Bastos TB, Jorgetti V, Rodolfo de Paula V, Teodoro WR, Lopes F (2020). The deleterious effects of smoking in bone mineralization and fibrillar matrix composition. Life Sci.

[37] Callaway DA, Jiang JX (2015). Reactive oxygen species and oxidative stress in osteoclastogenesis, skeletal aging and bone diseases. J Bone Miner Metab.

[38] Çetin A, Muhtaroglu S, Saraymen R, Öztürk A, Muderris I (2009). Smoking-induced bone defects may be due to oxidative damage in postmenopausal women. Turkey Clinics J Med Sci.

[39] Duthie GG, Arthur JR, James WP (1991). Effects of smoking and vitamin E on blood antioxidant status. Am J Clin Nutr.

[40] Ejaz S, Lim CW (2005). Toxicological overview of cigarette smoking on angiogenesis. Environ Toxicol Pharmacol.

[41] Ma L, Chow JY, Cho CH (1999). Cigarette smoking delays ulcer healing: role of constitutive nitric oxide synthase in rat stomach. Am J Physiol.

[42] Raines AL, Olivares-Navarrete R, Wieland M, Cochran DL, Schwartz Z, Boyan BD (2010). Regulation of angiogenesis during osseointegration by titanium surface microstructure and energy. Biomaterials.

[43] Park HS, Lee YJ, Jeong SH, Kwon TG (2008). Density of the alveolar and basal bones of the maxilla and the mandible. Am J Orthod Dentofacial Orthop.

[44] Di Stefano DA, Arosio P, Pagnutti S, Vinci R, Gherlone EF (2019). Distribution of trabecular bone density in the maxilla and mandible. Implant Dent.

[45] Eskandarloo A, Arabi R, Bidgoli M, Yousefi F, Poorolajal J (2019). Association between marginal bone loss and bone quality at dental implant sites based on evidence from cone beam computed tomography and periapical radiographs. Contemp Clin Dent.

[46] Chrcanovic BR, Albrektsson T, Wennerberg A (2017). Bone quality and quantity and dental implant failure: a systematic review and meta-analysis. Int J Prosthodont.

